# Editorial: Social and affective domain in home language development and maintenance research, volume II

**DOI:** 10.3389/fpsyg.2026.1813132

**Published:** 2026-03-13

**Authors:** Anastassia Zabrodskaja

**Affiliations:** Baltic Film, Media and Arts School, Tallinn University, Tallinn, Estonia

**Keywords:** family language policy (FLP), heritage language (HL), home language, language maintenance, language transmission, majority language, multilingualism, translanguaging

## 1 Introduction

The second volume of *Social and affective domain in home language development and maintenance research* continues to advance a crucial shift in multilingualism research: from viewing home language development primarily as a cognitive or structural phenomenon to understanding it as deeply embedded in emotional, relational, and societal processes. This ecological and relational perspective resonates with Bronfenbrenner's (1979) ecological systems theory, which conceptualizes development as emerging from dynamic interactions across nested social environments, and with subsequent work emphasizing the role of identity and investment in language learning (Darvin and Norton, 2015). Across diverse contexts—including migrant, refugee, Indigenous, and diaspora communities—this Research Topic foregrounds how affect, identity, agency, wellbeing, and communication ecologies shape language maintenance and transmission. The contributions gathered in this volume span family language policy, educational transitions, digital mediation, intergenerational relationships, cultural artifacts, and societal structures. Together, they demonstrate that home language maintenance is not merely a private family endeavor but a socially distributed, emotionally charged, and structurally conditioned process.

## 2 Contributions in this Research Topic

### 2.1 Emotional labor, identity, and intergenerational bonds

Several contributions illuminate the central role of emotional investment in heritage language (HL) transmission. Rokita-Jaśkow and Panek reveal the emotional labor of Polish migrant mothers who experience pride, responsibility, and anxiety while maintaining Polish in both exogamous and endogamous families. Their study highlights how intergenerational ties—especially with grandparents—serve as powerful affective motivators, while guilt and integration concerns create additional pressure. Similarly, Bilgory-Fazakas and Armon-Lotem show how Hungarian-speaking immigrant families in Israel sustain HL maintenance through strong cultural commitment and intergenerational communication. Bohnacker and Haddad document Arabic maintenance in Sweden, where parental confidence and supportive multilingual policies coexist with strong child agency. Fatima and Nadeem extend the intergenerational lens to Pakistan, demonstrating how multigenerational households, rituals, and emotional belonging reinforce HL transmission, even as dominant language pressures reshape younger generations' preferences. These findings further align with established frameworks of family language policy, which conceptualize language practices, beliefs, and management as interconnected dimensions shaping intergenerational transmission (Spolsky, 2009). Habak et al. broaden the family focus by examining fathers' wellbeing and self-efficacy in relation to home literacy engagement, highlighting the importance of paternal roles in sustaining affective and literacy practices within multilingual homes. Across these studies, HL maintenance emerges as sustained through emotional attachment, relational continuity, and identity affirmation—yet often accompanied by invisible labor and uneven responsibility. The recurring theme of emotional labor in these studies echoes Hochschild's (1983) foundational conceptualization of emotion work, underscoring how the maintenance of heritage languages often entails sustained affective management that remains socially undervalued and unevenly distributed within families.

### 2.2 Migration, age, and motivational adaptation

Another set of contributions examines how language trajectories intersect with migration timing, educational transitions, and motivational dynamics. Wang and Zhang demonstrate how age at migration shapes 1.5-generation Chinese immigrants' language outcomes in Australia, yet emphasize that family support, schooling, and personal motivation mediate attrition and maintenance. Zhuang and Wang analyze students' motivational adjustment during the transition from junior to senior high school, showing how instructional design, self-worth concerns, and perceived task difficulty affect engagement in English grammar learning. Wang and Wang investigate how foreign-language–related social grooming on social media enhances enjoyment through social capital and support, while privacy concerns moderate these benefits. Together, these studies underscore that language development is dynamically shaped by affective adaptation, institutional contexts, and evolving self-perceptions rather than by structural factors alone.

### 2.3 Digitalization, masspersonal communication, and media ecologies

Digital environments are playing an increasingly central role in shaping home language practices. Chen and Liu propose a masspersonal communication model that conceptualizes home language maintenance as emerging at the intersection of interpersonal, mass, and digitally networked contexts. Their framework highlights how digital platforms blend intimacy with public visibility, reshaping affective attachment and identity construction. Bloch's micro-interactional study of WhatsApp messaging in Russian–Hebrew bilingual families demonstrates how translanguaging practices construct multilingual familylects and reinforce intimacy and belonging. Wang and Wang's findings on social media engagement, alongside Zu et al.'s analysis of screen exposure and parental mediation in early literacy, further illustrate how digital practices can either enhance or undermine language development depending on guidance, privacy boundaries, and parental involvement. Collectively, these contributions show that digitalization is not peripheral but structurally embedded in contemporary family language ecologies.

### 2.4 Materiality, culture, and symbolic mediation

Language maintenance is also sustained through material and symbolic resources. Protassova and Yelenevskaya demonstrate how toys function as cultural artifacts in Russophone immigrant families, fostering creativity, emotional continuity, and intergenerational ties. Material objects—such as traditional dolls or toys brought from the country of origin—serve as anchors of memory and identity. Qi and You, examining exposure to foreign literature, show how narrative immersion and cultural empathy foster positive out-group attitudes, positioning literature as a form of indirect intercultural contact. These studies expand the analytical lens beyond spoken interaction, revealing how affective and cultural transmission operates through objects, narratives, and symbolic engagement.

### 2.5 Educational equity, policy, and societal responsibility

A final group of contributions situates home language development within broader institutional and policy frameworks. Inan and Harris argue that HL maintenance must be treated as a shared societal responsibility, advocating ecological collaboration among families, schools, policymakers, and communities. Song's scoping review of Chinese heritage language research in Inner Circle countries identifies conceptual gaps, terminological imprecision, and limited attention to non-Mandarin varieties, calling for more inclusive research and policy approaches. Burr highlights learner agency in Latvian diaspora classrooms, emphasizing the need for more student-centered pedagogies to strengthen engagement and literacy development. Nurhaliza et al. propose the culturally adaptive On–Off School Model for the Bajo sea-nomad community, aligning education with Indigenous lifeways and challenging rigid schooling structures. Figueiredo and Hefter underscore the importance of culturally sensitive translation practices in PTSD assessment for Ukrainian refugees, demonstrating how semantic shifts in emotion-related terminology can undermine diagnostic reliability. Together, these studies demonstrate that home language development cannot be sustained without structural support, culturally responsive policy, and institutional recognition of multilingualism as a public good.

### 2.6 Cross-cutting themes: affect, agency, and ecology

While the description of the contributions in this Research Topic is organized thematically, several cross-cutting dimensions connect them conceptually. First, affect consistently emerges as both a resource and a constraint. Emotional attachment, pride, empathy, enjoyment, and wellbeing function as enabling forces in language maintenance, yet emotional strain, anxiety, and structural pressure complicate family efforts. Across contexts, affect is not peripheral but constitutive of language development. Second, agency operates at multiple levels. This multilevel conceptualization of agency reflects social cognitive perspectives that view human action as situated yet agentive (Bandura, 2001), while sociocultural approaches emphasize the mediated and relational nature of agency in multilingual development. Children demonstrate linguistic agency in sibling interactions and classroom negotiations; parents exercise strategic agency through family language policy and media mediation; educators shape motivational trajectories through pedagogical design; and institutions either enable or constrain multilingual practices through policy decisions. Agency, however, is always situated within broader ideological and structural conditions. Third, an ecological perspective permeates the volume. Home language maintenance unfolds at the intersection of interpersonal relationships, digital environments, educational institutions, material culture, and sociopolitical structures. The findings collectively reinforce the view that no single domain—family, school, or media—can be analyzed in isolation. Rather, language development is the outcome of dynamic interactions across overlapping systems. This integrative lens underscores the necessity of interdisciplinary and multilevel approaches to the social and affective dimensions of multilingualism.

## 3 Practical and pedagogical implications

Across the contributions, several practical insights emerge for families, educators, and community practitioners. First, intentional and emotionally attuned family engagement remains central. Studies in this volume demonstrate that shared reading, cultural rituals, thoughtful media mediation, and sustained interaction in the heritage language significantly support language maintenance and early literacy. Importantly, parental wellbeing and self-efficacy—including fathers' confidence in their caregiving roles—directly influence children's literacy-related engagement. Second, pedagogical design matters. Research on motivational adaptation and learner agency suggests that concept-focused instruction, explicit scaffolding, and opportunities for student voice can strengthen engagement and linguistic confidence. In diaspora and heritage classrooms, systematically fostering learner agency rather than relying on sporadic flexibility appears crucial. Third, digital practices require guidance rather than prohibition. Social media interaction can enhance language enjoyment and social capital when privacy boundaries and active engagement are respected. Likewise, screen exposure in early childhood is not inherently detrimental; its impact depends heavily on parental mediation strategies and the displacement—or enrichment—of language-rich interaction. Together, these findings call for pedagogical approaches that are affect-sensitive, culturally responsive, and interactionally rich.

## 4 Policy and institutional perspectives

Beyond the family and classroom, this volume highlights the importance of structural and institutional support for multilingualism. Inan and Harris explicitly argue that heritage language maintenance must be reframed as a shared societal responsibility. Schools, policymakers, and community organizations play a decisive role in legitimizing multilingualism through inclusive curricula, dual language programs, teacher training, and equitable policy frameworks. This orientation resonates with Ruíz's (1984) influential distinction between viewing language as a problem, a right, or a resource—an ideological framing that remains central to contemporary debates on multilingual education and equity. Song's review further demonstrates that research and policy must move beyond homogenizing labels and recognize linguistic diversity within broad categories such as “Chinese,” ensuring visibility and support for underrepresented varieties. Nurhaliza et al.'s culturally adaptive On–Off School Model challenges rigid schooling structures and exemplifies how institutional flexibility can promote inclusion for mobile and Indigenous communities. Figueiredo and Hefter's work on PTSD assessment underscores that linguistic accuracy and cultural sensitivity in institutional tools are essential for equitable access to psychological services. Across these contributions, a consistent message emerges: sustainable home language development requires systemic collaboration. Without supportive educational structures, culturally sensitive institutional practices, and coherent policy commitment, the emotional and practical burden remains disproportionately on families.

## 5 Concluding remarks

Volume II deepens our understanding of the social and affective foundations of home language development in three significant ways. First, it foregrounds emotional labor, wellbeing, and identity as central—not peripheral—to language maintenance. Second, it integrates digital and masspersonal communication into the analysis of family language policy and intergenerational bonding. Third, it situates home language development within broader ecological systems, emphasizing shared societal responsibility. Such an ecological and justice-oriented perspective calls for continued integration of identity, ideology, and structural analysis into research on multilingual families, ensuring that affective and relational dimensions remain analytically central rather than peripheral. Across contexts as varied as migrant Europe, Australia, Pakistan, Israel, Sweden, Finland, Indonesia, Abu Dhabi, and diaspora communities worldwide, the contributions in this volume collectively argue that sustaining linguistic diversity requires emotional recognition, relational commitment, pedagogical innovation, and structural collaboration. By bringing together empirical, theoretical, and conceptual advances, this Research Topic invites scholars to adopt more ecologically grounded and affect-sensitive approaches to multilingual family life—and calls upon institutions to move beyond symbolic endorsement toward meaningful support of linguistic diversity in homes and communities worldwide.

## 6 Future research directions

The studies in this volume also point toward several promising avenues for future inquiry. First, greater attention is needed to underrepresented linguistic communities and varieties, as highlighted by the critique of homogenizing language labels. Expanding research beyond dominant or prestige varieties will allow for more nuanced understandings of linguistic identity and cultural continuity. Second, the rapid transformation of digital communication calls for longitudinal and mixed-method investigations into how masspersonal practices reshape intergenerational bonding, language ideology, and literacy development over time. The affective consequences of digital mediation—both positive and negative—remain an important area for systematic exploration. Third, more research is required on the distribution of emotional labor within families, including fathers' roles and children's agency, in order to move beyond mother-centered narratives of heritage language maintenance. Fourth, comparative and policy-oriented research can further illuminate how institutional frameworks either alleviate or intensify the burden placed on families. Empirical evaluation of educational models, community initiatives, and culturally adaptive schooling approaches would significantly advance the field. By pursuing these directions, future scholarship can continue to deepen our understanding of multilingual family life as an emotionally grounded, socially embedded, and structurally mediated phenomenon.
